# Efficacy and safety of omega-3-enriched lickable treats as adjunctive therapy for feline chronic gingivostomatitis: A randomized controlled trial

**DOI:** 10.14202/vetworld.2025.2344-2356

**Published:** 2025-08-18

**Authors:** Panithi Sukho, Sekkarin Ploypetch, Chakkarin Satthathum, Phirom Prompiram, Warunya Chakritbudsabong

**Affiliations:** 1 Department of Clinical Sciences and Public Health, Faculty of Veterinary Science, Mahidol University, Nakhon Pathom, Thailand; 2 Surgery Unit, Kasetsart University Veterinary Teaching Hospital, Faculty of Veterinary Medicine, Kasetsart University, Bangkok, Thailand; 3 The Monitoring and Surveillance Center for Zoonotic Disease in Wildlife and Exotic Animals (MoZWE), Faculty of Veterinary Science, Mahidol University, Nakhon Pathom, Thailand; 4 Department of Pre-clinic and Applied Animal Science, Faculty of Veterinary Science, Mahidol University, Nakhon Pathom, Thailand

**Keywords:** adjuvant therapy, cat oral health, cytokines, feline chronic gingivostomatitis, green-lipped mussel oil, inflammation, krill oil, nutraceuticals, omega-3 polyunsaturated fatty acids, lickable cat treat

## Abstract

**Background and Aim:**

Feline chronic gingivostomatitis (FCGS) is a debilitating inflammatory condition of the oral cavity in cats, associated with chronic pain, salivation, halitosis, and reduced quality of life. Omega-3 polyunsaturated fatty acids (n-3 PUFAs) have known anti-inflammatory properties and may offer a supportive treatment option. This study aimed to evaluate the safety and clinical efficacy of two marine-derived n-3 PUFA sources – krill oil and green-lipped mussel (GLM) oil – administered through lickable cat treats in cats with FCGS.

**Materials and Methods:**

Sixteen FCGS cats were randomized into three double-blinded treatment groups to receive daily lickable treats: Control (no n-3 PUFA), krill oil (100 mg), or GLM oil (100 mg) for 28 days. Clinical outcomes (stomatitis and pain scores, halitosis, and salivation) were assessed on days 0, 14, and 28 by veterinarians. Owner-reported outcomes and inflammatory cytokine levels (interleukin 6, interleukin 1 beta, and interferon-gamma) were also evaluated.

**Results:**

Both krill and GLM oil treatments were well-tolerated, with no adverse changes in body weight, hematological, or biochemical parameters. Mean stomatitis and pain scores showed a non-significant downward trend in all groups. Halitosis and salivation improved in seven cats, particularly in the GLM and control groups. Owner-reported improvement was observed in 10 of 16 cats, with the highest in the krill oil group (75%). Cytokine levels demonstrated high variability and no statistically significant changes. No significant differences were observed between the n-3 PUFA sources.

**Conclusion:**

Daily administration of n-3 PUFA-enriched cat treats is safe and may provide mild clinical benefit in cats with FCGS, particularly in alleviating oral discomfort. While no significant differences were found between krill and GLM oils, both formulations were palatable and suitable as adjunctive therapy. Further research with larger cohorts, extended durations, and sensitive scoring systems is warranted to optimize treatment protocols.

## INTRODUCTION

Feline chronic gingivostomatitis (FCGS) is a painful, erosive inflammatory condition characterized by lesions affecting the gingiva, buccal mucosa, and caudal oral cavity in cats [1, 2]. It affects approximately 26% of domestic cats [3] and 39% of those presenting with oral lesions [4], with no age predilection. Clinical signs commonly include ptyalism, oral pain, pawing at the mouth, halitosis, intraoral bleeding, dysphagia, weight loss, and poor grooming. FCGS significantly impairs feline quality of life and poses substantial treatment challenges. Many cats require long-term management, and some ultimately face euthanasia due to refractory disease [1, 2]. The etiology of FCGS is multifactorial, inv-olving genetic predisposition, environmental stressors, microbiome dysbiosis, nutritional imbalances, and viral infections [5–7]. These factors disrupt immune homeostasis and drive chronic mucosal inflammation. Elevated levels of inflammatory cytokines such as interleukin (IL)-16, IL-10, and interferon alpha have been detected in affected cats [8, 9]. Despite extensive research, the pathogenesis remains incomp-letely understood [1, 2]. Current treatments include anti-inflammatory drugs, immunosuppressants, analge-sics, and antibiotics. In severe or non-responsive cases, full or partial dental extractions are commonly employed. However, pharmacologic therapy alone often yields limited long-term success and may pose risks in older cats or those with renal disease or viral infections [2, 10–12]. Although surgical intervention can result in partial or complete remission in approximately 70% of cases, it is invasive, costly, and may not be universally accessible [11, 13]. Emerging therapies – such as recombinant feline interferon omega [14, 15], bovine lactoferrin combined with piroxicam [16], and mesenchymal stem cell therapy [17] – have shown promise in refractory cases. However, these options are often expensive, require specialized facilities, and may be unavailable in remote areas. Therefore, a safe, cost-effective, and minimally invasive adjunct therapy would be highly beneficial, particularly for cats undergoing surgical management or those unresponsive to conventional treatment.

Omega-3 polyunsaturated fatty acids (n-3 PUFAs), including eicosapentaenoic acid (EPA) and docosahexaenoic acid (DHA), are known for their anti-inflammatory and immunomodulatory properties [18]. In human medicine, n-3 PUFAs have demonstrated efficacy in managing esophageal and oral inflammatory conditions [19, 20]. In veterinary practice, they are commonly used to treat inflammatory disorders such as allergic dermatitis and osteoarthritis in both dogs and cats [21, 22]. In cats, the role of n-3 PUFAs in oral inflammation remains underexplored. Only a few studies have evaluated their potential benefits for stomatitis [23] and periodontal disease [24, 25]. A prior study showed that cats with FCGS receiving diets with n-6:n-3 PUFA ratios of 10:1 or 40:1 after molar and premolar extraction exhibited improvements in stomatitis scores and cytokine levels, although these changes were not statistically significant [23]. Evidence in this area is mostly limited to case reports or safety data in healthy animals [26], with a lack of controlled trials or standardized dosing regimens for FCGS. EPA and DHA influence mucosal immune responses through several mechanisms [27, 28]. These include incorporation into cellular membranes, where they modulate fluidity and lipid raft composition, thereby altering intracel-lular signaling and downregulating pro-inflammatory mediators [27, 29, 30]. In addition, n-3 PUFAs shift lipid mediator synthesis toward anti-inflammatory profiles and enhance phagocytic activity and adaptive immunity by modulating T- and B-cell responses [27, 28, 30, 31].

Despite these immunological benefits, maintain-ing a balanced dietary intake of omega-3 and omega -6 PUFAs is crucial. An excess of omega-3 fatty acids may lead to immune suppression, underscoring the importance of maintaining an optimal n-6:n-3 ratio [32]. Marine sources of n-3 PUFAs – including fish, krill, and green-lipped mussel (GLM) oils – have demonstrated superior anti-inflammatory efficacy in cats compared to plant-based sources [25, 33]. GLM; *Perna canaliculus* oil, a marine-derived n-3 PUFA source, has been shown to be safe for feline use at doses up to 300 mg daily for 28 days [34]. Case reports have described its use as adjunct therapy in cats with conditions such as chronic renal failure with cystitis [35] and chronic juvenile gingivitis [36]. More recently, krill oil extracted from *Euphausia supe-rba* has emerged as a novel and sustainable n-3 PUFA source, also containing choline and xanthine, which may offer additional health benefits [37]. Krill oil has demonstrated anti-inflammatory effects in animal models of osteoarthritis [38], ulcerative colitis [39], and liver inflammation [40]. In comparative studies on canine cartilage degradation, krill oil outperfor- med both fish and GLM oils [41]. However, its therapeutic impact in cats with FCGS remains unexplored.

Despite FCGS being a debilitating and increas-ingly recognized condition in feline medicine, current treatment approaches remain largely inadequate. While anti-inflammatory medications and surgical dental extractions form the cornerstone of FCGS management, these strategies often present signifi-cant limitations, including incomplete resolution, relapse, and adverse effects – particularly in geriatric or immunocompromised cats. To date, only one interventional study and two reviews have addressed their role in feline oral inflammation, and no studies have directly compared the efficacy of different marine-derived n-3 PUFA sources in cats with naturally occurring FCGS. Furthermore, the dose-response relationship, safety profile, and immunomodulatory effects of krill oil and GLM oil in FCGS remain uncharacterized. Therefore, there is a clear lack of controlled, head-to-head trials evaluating the therapeutic potential of these marine-sourced n-3 PUFAs in this context.

This study aimed to address the identified research gaps by evaluating the clinical efficacy and safety of two marine-derived sources of omega-3 fatty acids – krill oil and GLM oil – formulated into lickable cat treats, as adjunctive treatments for FCGS. Specifically, the study sought to:

- Assess whether daily supplementation with krill oil or GLM oil for 28 days can alleviate clinical signs associated with FCGS, including oral pain, salivation, halitosis, and mucosal inflammation
- Compare the therapeutic effects between krill oil and GLM oil, thereby identifying any differential anti-inflammatory efficacy between these sources
- Evaluate the impact of supplementation on inflammatory cytokine profiles (IL-6, IL-1β, and interferon-gamma [IFN-γ]), hematological para-meters, and general health status to establish safety
- Examine owner-reported outcomes to determine acceptability and perceived clinical benefit in a real-world setting.

Through this investigation, we aimed to determine whether omega-3-enriched cat treats could serve as a safe, palatable, and cost-effective adjunctive therapy for managing FCGS in clinical practice – especially in cases where conventional medical or surgical approaches are insufficient or contraindicated.

## MATERIALS AND METHODS

### Ethical approval

All procedures involving animals were reviewed and approved by the Institutional Animal Care and Use Committee of the Faculty of Veterinary Science, Mahidol University, Thailand (Approval ID: MUVS-2019-08-42).

### Study period and location

Sample collection was conducted from 15 November 2019 to 24 April 2021. Samples were sour-ced from Prasu-Arthorn Small Animal Hospital, Mahidol University, Nakhon Pathom, and various private animal hospitals and clinics located in Bangkok, Nonthaburi, and Nakhon Pathom, Thailand. All laboratory testing and data analysis were performed at the Faculty of Veterinary Science, Mahidol University, Nakhon Pathom, Thailand.

### Preliminary safety evaluation in healthy cats

To evaluate the safety of krill oil supplementation in feline diets, five healthy indoor cats were enrolled in a 28-day preliminary tolerability trial. Each cat received a lickable treat containing 100 mg of krill oil daily. Clinical assessments, including body weight measurements and physical examinations, were conducted on Days 0, 3, and 28. Blood samples were collected on these days to assess hematological and serum biochemical parameters, including total protein, albumin, alanine aminotransferase (ALT), alkaline phosphatase (ALP), aspartate aminotransferase (AST), creatinine (CREA), and blood urea nitrogen (BUN). No adverse effects were observed during the trial.

### Study design and enrolment

This pilot clinical trial adopted a randomized, placebo-controlled, double-blind design. The target sample size was 18 cats, based on available resources and subject eligibility to support proof-of-concept evaluation. Client-owned indoor cats aged 1–14 years of any breed or sex, diagnosed with naturally occurring FCGS, were screened for enrollment.

Eligibility was confirmed through physical exa-mination and laboratory analysis to rule out systemic illness or renal/hepatic dysfunction. Cats receiving corticosteroids or immunosuppressive drugs were excluded unless such treatments had been discontinued at least 2 weeks prior. Cats with manageable systemic comorbidities (e.g., anemia, feline immunodeficiency virus [FIV], and diabetes) were included and documented accordingly.

### Randomization and blinding

Eligible FCGS cats were randomly assigned to one of three treatment groups using a block randomization strategy. Allocation concealment was achieved through sequentially lettered, opaque, sealed envelopes. Both veterinarians and pet owners remained blinded to group assignments throughout the 28-day study period. All cats were maintained on a standardized commercial feline diet and received identical oral hygiene support during the trial.

### Preparation of lickable cat treats and fatty acid profiling

Three formulations of lickable cat treats were prepared:

- Control group: Chicken and fish oil-based treats with no added n-3 PUFAs
- Krill oil group: Control formulation + 100 mg krill oil
- GLM oil group: Control formulation + 100 mg GLM oil (PCSO-524®).

Each 15-g sachet was visually indistinguishable and coded to preserve blinding. Fatty acid profiling was conducted on one sachet per group at the Kasetsart University Institute of Food Research and Product Development to confirm n-3 PUFA content.

### Clinical monitoring of FCGS cats

**Figure 1:**
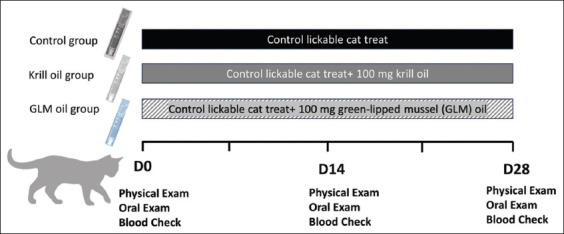
Clinical trial diagram for this experiment in cats with feline chronic gingivostomatitis.

Veterinarians conducted standardized evaluations on Days 0, 14, and 28. These assessments included body weight, vital signs, auscultation, capillary refill time, skin turgor, abdominal palpation, and comprehensive oral examination (Figure 1). Oral health parameters included inflammation of the gingiva, buccal mucosa, palatoglossal folds, tongue, and caudal oral cavity. Stomatitis was graded using a 0–3 severity scale. Pain scores were recorded using a validated veterinary pain scale (Table 1). Additional signs such as halitosis, ptyalism, and lymphadenopathy were documented (Supplementary Tables 1 and 2).

**Table 1:** Clinical scoring system for veterinarian-assessed oral pain in cats.

Score	Pain level	Clinical signs
0	No pain	- Bright, alert, and responsive with a normal attitude
		- No spontaneous vocalization
		- Allows the mouth to be opened easily without resistance or signs of discomfort.
1	Mild pain	- Normal attitude but may exhibit slight sensitivity to palpation around the mouth
		- Does not show aggression when the mouth is opened but shows sensitivity when inflamed areas are touched
		- Can hold the mouth open for more than 10 s.
2	Moderate pain	- May exhibit spontaneous or consistent vocalization
		- Sensitive to touch around the mouth - Requires physical restraint to open the mouth and may vocalize during the procedure
		- Can only hold mouth open for 5–10 s.
3	Severe pain	- Very sensitive to any touch around the mouth
		- Vocalizes on restraint and displays aggressive behaviors (e.g., hissing and attempting to bite)
		- Can only hold mouth open for 1–2 s or does not permit veterinarians to open, touch, or clean the mouth.

### Owner-based observations

Cat owners completed standardized daily obse-rvation forms, which included ordinal scales for appearance, food intake, behavior (e.g., grooming/playfulness), pain response, salivation, and halitosis. These observations were translated into total scores (maximum = 5) on Days 0, 14, and 28, with changes in scores used to assess clinical improvement.

### Laboratory analysis

Venous blood samples collected on Days 0, 14, and 28 were analyzed for complete blood cell counts (CBCs) and serum biochemical markers (ALT, ALP, AST, CREA, BUN). Serum was centrifuged and stored at –80°C until cytokine analysis.

Proinflammatory cytokine concentrations (IL-6, IL-1β, and IFN-γ) were quantified using feline-specific DuoSet enzyme-linked immunosorbent assay kits (R&D Systems, USA) according to the manufacturer’s instructions. Detection limits were 62.5–4,000 pg/mL for IFN-γ and 31.3–2,000 pg/mL for both IL-6 and IL-1β.

### Statistical Analysis

All statistical analyses were conducted using NCSS software (NCSS, LLC, Kaysville, Utah, USA, version 12.0.11). For the safety study in healthy cats, repeated measures were analyzed using a generali- zed linear model. For the clinical trial, stomatitis scores, pain scores, cytokine concentrations, and serum chemistry data were analyzed using a generali-zed linear model with non-parametric, distribution-free repeated measures blocked by individual cat.

Group comparisons for age, body weight, and dise-ase duration were performed using the Kruskal-Wallis test, while Fisher’s exact test assessed differences in sex, breed, and stomatitis grade. The Chi-square test evaluated owner-reported satisfaction across treatment groups. Statistical significance was defined as p < 0.05, and data are presented as mean ± standard error of the mean.

## RESULTS

### Fatty acid profile of lickable cat treats

The fatty acid composition of the lickable treats varied among treatment groups. The highest n-3 PUFA content was found in the GLM oil group (1.27% of dry matter), followed by the krill oil group (1.0%) and the control group (0.8%) (Table 2). Correspondingly, the n-6:n-3 PUFA ratios were 7:1 for the GLM oil group, 9:1 for the krill oil group, and 11:1 for the control group. These differences reflect a more favorable anti-inflammatory profile in the marine-supplemented groups.

**Table 2:** Results of fatty acid analysis and comparison of n-3 PUFAs and n-6 PUFAs in each group.

Analysis	Control	% of the DM	

		Krill oil	GLM oil
Saturated fatty acid content			
Myristic acid	0.20	0.93	0.47
Palmitic acid	7.60	8.53	8.60
Stearic acid	2.20	2.33	2.53
Lignoceric acid	0.07	0.27	0.73
Unsaturated fatty acid content			
Palmitoleic acid	1.33	1.73	1.60
cis-9-Oleic acid	13.00	13.87	18.93
cis-11-Eicosenoic acid	0.20	0.20	0.27
Linoleic acid	8.67	8.93	8.67
gamma-Linolenic acid	0.07	0.07	0.07
alpha-Linolenic acid	0.73	0.87	0.80
Arachidonic acid	0.27	0.27	0.27
Docosahexaenoic acid	0.07	0.20	0.47
n-3 PUFAs (Omega-3)	0.80	1.00	1.27
n-6 PUFAs (Omega 6)	9.00	9.27	9.00
Total fat	36.13	40.13	45.53
Omega 6:3 ratio	11.3:1	9.3:1	7.1:1

n-3 PUFAs=Omega-3 polyunsaturated fatty acids, n-6 PUFAs=Omega-6 polyunsaturated fatty, DM=Dry matter, GLM=Green-lipped mussel, PUFAs=Polyunsaturated fatty acids

A 28-day safety evaluation in five healthy cats fed krill oil-supplemented treats showed no adverse changes in body weight or serum biochemical parameters. In addition, improved coat quality (shinier and softer fur) was observed in all cats by the end of the trial.

### Clinical outcomes in FCGS cats receiving supplemented treats

Nineteen cats were initially enrolled, but three were excluded due to unrelated illness (n = 1), loss to follow-up (n = 1), or refusal to consume the treats (n = 1), resulting in 16 cats completing the study. These included six cats in the control group, four in the krill oil group, and six in the GLM oil group (Table 3). The enrolled cats were aged 3–8 years and of both sexes. All cats were domestic short-hair (DSH), with an average age of 5.3 ± 1.9 years. Minor comorbidities such as anemia, diabetes mellitus, and retroviral infections (FIV/Feline leukemia virus-positive) were documented in some cases.

**Table 3:** Signalments and groups of cats included in the study.

Cat	Group	Age (years)	Sex	Breed	Systemic disease	FeLV/FIV	Stomatitis grade	BW (kg)	Length of the FCGS before the study (months)
1	Control	7	Mc	DSH	No	No	3	4.1	24
2	GLM oil	3	Mc	DSH	Diabetic	No	2	4.7	18
3	Control	7	Mc	DSH	No	No	2	2.4	60
4	GLM oil	8	Mc	DSH	No	FIV+	2	3	12
5	Krill oil	7	Mc	DSH	No	No	3	3.9	15
6	Control	5	Fs	DSH	No	No	2	3.9	4
7	GLM oil	8	Fs	DSH	Anemia	No	3	3.2	3
8	GLM oil	3	M	DSH	Anemia	FeLV+	2	4	5
9	Krill oil	4	Mc	DSH	No	No	1	5.8	24
10	Control	4	Fs	DSH	No	No	2	3.2	9
11	GLM oil	6	Fs	DSH	No	No	2	3.2	6
12	GLM oil	6	Fs	DSH	No	No	3	3	24
13	Krill oil	3	Fs	DSH	No	No	2	3.3	4
14	Krill oil	7	Fs	DSH	No	No	1	3.9	1
15	Control	5	Fs	DSH	No	No	2	3.6	3
16	Control	3	M	DSH	No	FeLV+	1	5.1	3

GLM=Green-lipped mussel, Mc=Male castrated, M=Male, Fs=Female sterile, DSH=Domestic short-hair cat, FIV+=Feline immunodeficiency virus-positive, FeLV+=Feline leukemia virus-positive, BW=Body weight

Blinding integrity was strictly maintained throu-ghout the study. All treatments were indistinguishable in appearance, and group allocation remained concealed from owners and veterinarians until after data analysis. No formal blinding assessment was conducted, but procedural consistency was ensured.

**Figure 2:**
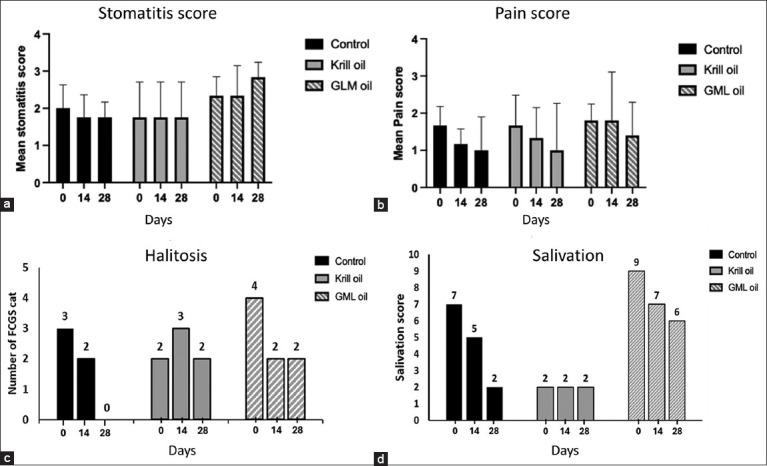
Clinical outcome of feline chronic gingivostomatitis cats based on veterinarians’ evaluations on days 0, 14, and 28. (a) Mean stomatitis score (0–3), (b) Mean pain score (0–3), (c) Halitosis cat, and (d) Overall salivation score (0–2) of each group. Control (n = 6), Krill oil (n = 4), and GML oil (n = 6). GML=Green-lipped mussel.

At baseline (Day 0), mean stomatitis scores were comparable across groups: 2.00 (standard error [SE] = 0.51) in the control, 1.75 (SE = 0.94) in the krill oil group, and 2.00 (SE = 0.41) in the GLM group. Scores remained similar on Day 14 and Day 28. By Day 28, mean scores were 1.75 in the control, 1.75 in the krill oil, and 2.80 in the GLM group. No statistically significant changes in stomatitis severity were observed either within or between groups over time (p > 0.05) (Figure 2a; Table 4).

**Table 4:** Comparison of stomatitis score and pain score outcome at each time point after daily cat treatment.

Parameters	Group	Time point	Mean score	SE	95% CI		p-value

					Lower	Upper	
Stomatitis score	Control	Day 0	2	0.51	1.49	2.51	1.00
		Day 14	1.75	0.49	1.26	2.24	
		Day 28	1.75	0.33	1.42	2.08	
	Krill oil	Day 0	1.75	0.94	0.81	2.69	1.00
		Day 14	1.75	0.94	0.81	2.69	
		Day 28	1.75	0.94	0.81	2.69	
	GLM oil	Day 0	2.3	0.41	1.92	2.75	0.246
		Day 14	2.3	0.65	1.68	2.99	
		Day 28	2.8	0.33	2.51	3.16	
Pain score	Control	Day 0	1.67	0.41	1.25	2.08	0.209
		Day 14	1.17	0.33	0.84	1.49	
		Day 28	1.00	0.72	0.28	1.72	
	Krill oil	Day 0	1.75	0.94	0.81	2.69	0.829
		Day 14	1.5	0.57	0.93	2.07	
		Day 28	1.25	1.47	−0.22	2.72	
	GLM oil	Day 0	1.67	0.41	1.25	2.08	0.448
		Day 14	1.33	0.97	0.36	2.3	
		Day 28	1.00	0.72	0.28	1.72	

CI=Confidence interval, SE=Standard error, GLM=Green-lipped mussel

**Figure 3:**
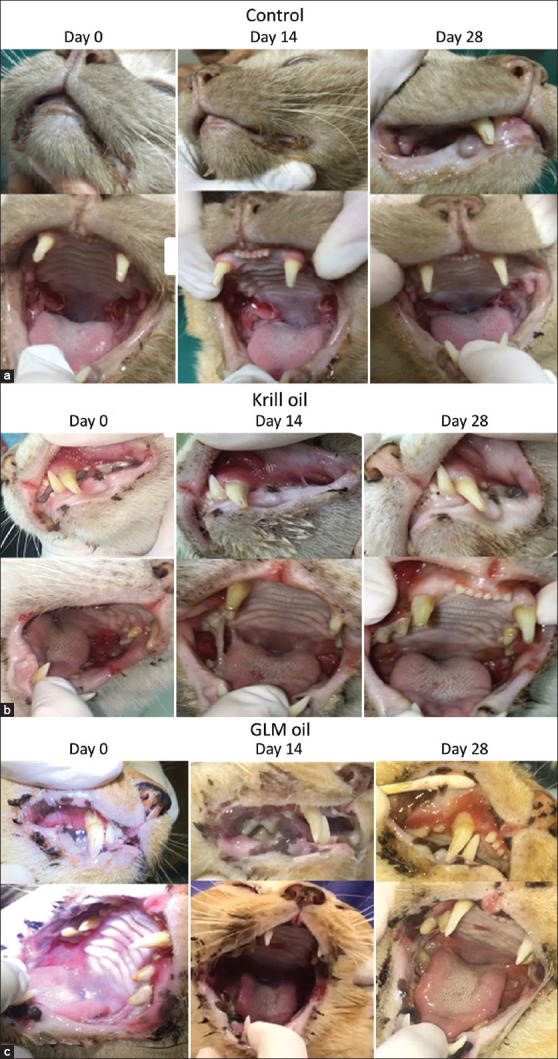
Clinical outcomes of cats with feline chronic gingivostomatitis based on veterinarians’ evaluations on days 0, 14, and 28. (a) Control group, (b) Krill oil group, and (c) GML oil group. GML=Green-lipped mussel.

A non-significant trend toward pain reduction was observed by Day 14 in the control and krill oil groups, with all groups showing lower pain scores by Day 28. Day 0 mean pain score was 1.67 in control group, 1.75 in Krill oil group, and 1.67 in GLM group, a non-significant decreasing in mean pain scores was observed at Day 14 in the control (1.17, p = 0.20, 95%CI [0.84–1.4]), krill oil groups (1.5,p = 0.82, 95%CI [0.93–2.07]), and across all groups by Day 28 (1.00, 1.25, and 1.00), respectively. (Figure 2b and Table 4). Halitosis was initially present in 9 of 16 cats. After 28 days of treatment, five cats showed resolution of halitosis, with improvements most notable in the control and GLM oil groups (Figure 2c). Twelve cats exhibited salivation at baseline, and eight of these showed improvement by Day 28. Reductions in salivation scores were observed primarily in the control and GLM oil groups (Figure 2d). Although these findings suggested clinical improvement, none reached statistical significance (p > 0.05) (Figure 3).

### Owner-reported clinical improvement

**Figure 4:**
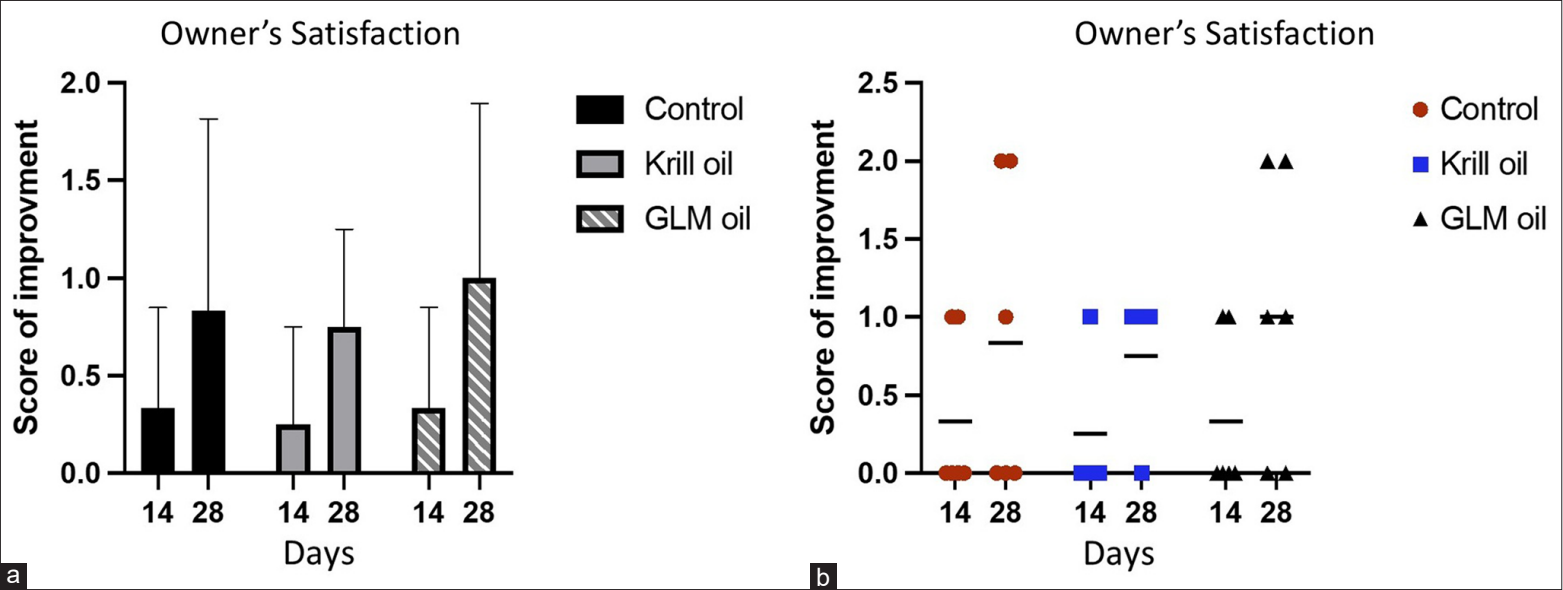
Owner evaluation results showing improvements during studies on feline chronic gingivostomatitis cats on days 14 and 28 compared with day 0. No improvement=score 0, mild improvement=score 1, significant improvement=score 2 and cure=score 3. (a) Mean improvement score (0–3) ± standard deviation (b) Individual plot of improvement score (0–3). Control (n = 6), Krill oil (n = 4), and GLM oil (n = 6). GML=Green-lipped mussel.

Owner-based ordinal scale assessments revealed that 10 of 16 cats showed improvement following the 28-day supplementation. Improvement was reported in 75% (3/4) of cats in the krill oil group, 66.7% (4/6) in the GLM group, and 50% (3/6) in the control group. However, the differences between groups were not statistically significant (p = 0.7, Cramér’s V = 0.21; 95% confidence interval: 0–0.55) (Figure 4).

### Hematological and cytokine analysis

**Figure 5:**
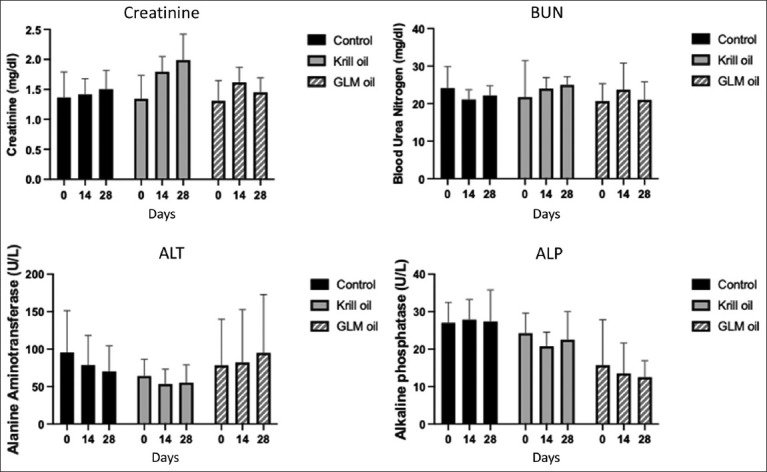
Blood chemistry results from feline chronic gingivostomatitis cats in each group; Control (n = 6), Krill oil (n = 4), and green-lipped mussel oil groups (n = 6). BUN=Blood urea nitrogen, ALT=Alanine aminotransferase, and ALP=Alkaline Phosphatase

No significant changes were observed in CBC, CREA, BUN, ALT, or ALP levels across the three treatment groups throughout the study (Figure 5 and Table 5).

**Table 5:** Hematology and biochemistry profiles of FCGS cats before (Day 0) and after (Day 4 and Day 28) receiving cat treats. Each value represents the mean ± standard error of the mean.

Parameters	Time point	Control	Krill oil	GLM oil	p-value between group	Normal range
White blood cell count (cell/µL)	Day 0	14,418 ± 7,217	28,003 ± 18,412	17,168 ± 5,282	0.16	5,500–19,500
	Day 14	13,307 ± 6,321	21,943 ± 17,765	13,900 ± 3,390	0.35	
	Day 28	12,858 ± 8,031	30,018 ± 29,421	14,748 ± 5,988	0.22	
	p-value	0.80	0.42	0.48	0.25	
Lymphocyte count (cell/µL)	Day 0	1,639 ± 646	2,955 ± 2,136	2,050 ± 537	0.24	1,500–7,000
	Day 14	1,611 ± 1,093	2,128 ± 1,204	1,872 ± 447	0.70	
	Day 28	1,442 ± 771	5,065 ± 5,981	1,882 ± 662	0.17	
	p-value	0.82	0.34	0.84	0.26	
Neutrophils count (cell/µL)	Day 0	11,741 ± 464	22,166 ± 15,838	12,935 ± 4,399	0.20	2,500–14,000
	Day 14	10,379 ± 5,134	17,273 ± 15,627	10,381 ± 2,968	0.19	
	Day 28	10,402 ± 7,323	21,524 ± 19,503	10,929 ± 4,816	0.26	
	p-value	0.77	0.45	0.51	0.29	
Creatinine (mg/dL)	Day 0	1.37 ± 0.4	1.34 ± 0.4	1.31 ± 0.3	0.97	0.2–2.1
	Day 14	1.42 ± 0.3	1.80 ± 0.3	1.62 ± 0.2	0.11	
	Day 28	1.50 ± 0.3	1.99 ± 0.4	1.45 ± 0.2	0.05	
	p-value	0.45	0.05	0.09	0.32	
Blood urea nitrogen (mg/dL)	Day 0	24.1 ± 5.7	21.8 ± 9.7	20.7 ± 4.7	0.66	10–30
	Day 14	21.1 ± 2.7	24 ± 2.9	23.7 ± 7.1	0.57	
	Day 28	22.1 ± 2.7	25 ± 2.2	21 ± 4.8	0.25	
	p-value	0.41	0.64	0.47	0.78	
Alanine aminotransferase (Unit/L)	Day 0	95.5 ± 55.8	64 ± 22.3	78.2 ± 61.7	0.65	20–100
	Day 14	78.5 ± 39.5	53.3 ± 20	82 ± 70.7	0.66	
	Day 28	70 ± 34.2	55.3 ± 23.6	94.8 ± 77.9	0.52	
	p-value	0.24	0.40	0.47	0.71	
Alkaline phosphatase (Unit/L)	Day 0	27 ± 5.4	24.3 ± 5.4	15.7 ± 12.1	0.10	10–90
	Day 14	27.8 ± 5.4	20.8 ± 3.8	13.5 ± 8.1	0.01*	
	Day 28	27.3 ± 8.5	22.5 ± 7.5	12.5 ± 4.4	0.01*	
	p-value	0.94	0.24	0.55	0.69	

* p < 0.05. FCGS=Feline chronic gingivostomatitis, GLM=Green-lipped mussel

**Figure 6:**
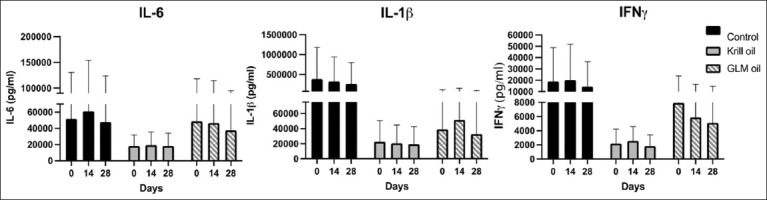
Serum cytokine levels of IL-6, IL-1β, and IFN-ɣ from enzyme-linked immunosorbent assay from feline chronic gingivostomatitis cats in the study on Day 0, 14, and 28. Control (n = 6), Krill oil (n = 4) and green-lipped mussel (GML) oil (n = 6). IL-6=Interleukin 6, IL-1β=Interleukin 1 beta, and IFN-ɣ=Interferon-gamma.

Serum cytokine concentrations (IL-6, IL-1β, and IFN-γ) were also evaluated at all time points. Although mean values varied slightly, there were no statistically significant differences in cytokine levels between groups or over time (Figure 6). Notably, several cytokine measurements were below the detection threshold in some cats, contributing to high standard deviations in group means (Supplementary Figure 2).

## DISCUSSION

### Safety and tolerability of omega-3 supplemented treats

This study evaluated the safety and therapeutic potential of marine-derived n-3 PUFA-enriched lickable treats in cats diagnosed with FCGS. Supplementation with 100 mg of krill oil or GLM oil (PCSO-524®) for 28 days was found to be well tolerated in both healthy and FCGS-affected cats. No adverse clinical signs or hematological abnormalities were observed, supporting the safety of short-term use. These findings align with previous reports on the safety of GLM and algal oil supplementation in felines [26, 34–36]. To date, no standardized toxic threshold or long-term dosing guidelines for n-3 PUFA use in cats have been established.

### Fatty acid profile and anti-inflammatory implications

Although krill oil has recently gained attention as a novel and sustainable source of n-3 PUFAs, its impact on feline inflammatory conditions, particularly FCGS, has not been previously studied. Experimental and clinical studies in murine and human models have demonstrated the anti-inflammatory potential of krill and GLM oils through the downregulation of proinflammatory mediators and oxidative stress pathways [39, 40]. The cat treats formulated in this study exhibited favorable n-6:n-3 ratios – 7:1 for GLM, 9:1 for krill oil, and 11:1 for the control. These values fall within the range associated with reduced systemic inflammation [42]. Despite this, no significant differen-ces in stomatitis scores were observed between groups. This outcome mirrors prior research in which FCGS cats receiving a 10:1 n-6:n-3 PUFA diet showed only modest clinical and cytokine changes post-extraction [23].

According to small animal nutritional guidelines, omega-3 content between 0.35% and 1.8% dry matter is recommended for skin inflammation manage-ment [43]. The GLM and krill oil groups in this study achieved 1.7% and 1.0% n-3 PUFA content, respecti- vely, while the control group reached 0.8%. The absence of a marked therapeutic effect could reflect the complex, multifactorial nature of FCGS, which may require higher doses or prolonged treatment for measurable clinical improvement.

### Clinical trends and observed outcomes

While stomatitis scores remained unchanged across all groups, a mild, non-significant reduction in pain scores was noted on Day 14 in the control and krill oil groups, and across all groups by Day 28. Improvements in halitosis and salivation – especially in the control and GLM oil groups – further suggested oral comfort benefits. These trends align with findings that cats without hypersalivation often exhibit reduced oral pain [44]. Notably, such improvements may reflect reduced mucosal pain rather than visible resolution of inflammation.

Although the anti-inflammatory efficacy of n-3 PUFAs may be lower than that of corticosteroids or NSAIDs, the absence of systemic adverse effects offers a compelling case for their use in cats with contraindi-cations to immunosuppressive drugs, such as those with viral infections. In addition, owners reported high palatability and ease of administration, with subjective improvements observed in 50%–75% of cases.

### Limitations in sensitivity and statistical power

The lack of statistically significant findings may be attributed to multiple methodological limitations. First, the sample size was modest (n = 16), limiting statistical power and increasing the risk of Type II error. Second, the clinical scoring systems employed – stomatitis severity and pain scores ranging from 0 to 3 – lacked granularity. While suitable for routine veterinary assessments, these scales may not detect subtle clinical changes. For future research, tools such as the Stomatitis Disease Activity Index (SDAI) and a 10-point pain scale may offer enhanced resolution and analytic sensitivity [8].

### Inflammatory cytokine trends and interpretation

Cytokine analysis revealed high interindividual variability, yet serum levels of IL-6, IL-1β, and IFN-γ trended downward in all groups by Day 28. However, none of these changes were statistically significant. This could be due to assay limitations, including detection thresholds (62.5–4,000 pg/mL for IFN-γ and 31.3–2,000 pg/mL for IL-6 and IL-1β), which may fail to detect subtle changes or low-level cytokine activity.

Preclinical studies have shown that n-3 PUFAs downregulate IL-1β gene expression [19], a trend echoed in our control and krill oil groups. Similarly, previous FCGS research reported reductions in infl-ammatory cytokines following high-dose EPA/DHA supplementation [23]. Another investigation on stem cell therapy in FCGS demonstrated increased IL-6 and decreased IFN-γ levels 3 months post-treatment [17]. While our study also found downward trends by Day 28, statistical confirmation was precluded by high variability.

### Implications for long-term use and future research

Lickable treats enriched with n-3 PUFAs may offer a safe, palatable adjunctive therapy to help alleviate oral discomfort in cats with FCGS. However, they should not exceed 10% of the cat’s daily caloric intake. In human periodontal studies, long-term supplementation with 2000 mg/day of EPA/DHA over 3–12 months has yielded significant reductions in inflammation [45]. Although our formulation contained a higher PUFA concentration (100 mg) than previous feline studies, the short 28-day duration may have been insufficient to yield measurable outcomes.

Extended trials of at least 6–12 weeks, as demonstrated in other models such as murine period-ontitis [46] and feline dermatologic inflammation [25], may be necessary to fully evaluate clinical benefits. Moreover, future studies should explore higher dosing strategies and standardized scoring systems in larger populations to clarify the therapeutic role of n-3 PUFAs in FCGS management.

## CONCLUSION

This study demonstrated that daily administration of lickable cat treats enriched with 100 mg of krill oil or GLM oil over a 28-day period was safe and well-tolerated in both healthy cats and those affected by FCGS. No adverse effects were observed on clinical parameters or blood biochemistry, and all cats maintained normal body condition and hematologic profiles throughout the study period. The fatty acid profiling confirmed a favorable n-6:n-3 PUFA ratio in the supplemented treats (7:1 for GLM and 9:1 for krill oil), which aligns with anti-inflammatory thresholds reported in previous studies.

Although the supplemented groups did not achieve statistically significant improvements in stomatitis or pain scores, non-significant clinical trends were obs-erved. Specifically, reductions in oral pain, halitosis, and hypersalivation were noted by Day 28, and owner-reported outcomes indicated that up to 75% of cats experienced some degree of improvement – particularly in the krill oil group. In addition, inflammatory cytokine levels (IL-6, IL-1β, and IFN-γ) showed a downward trend, despite high interindividual variability and limited statistical power.

From a practical perspective, these findings suggest that marine-derived n-3 PUFA supplementation, particularly in the form of a highly palatable treat, may serve as a feasible adjunctive therapy for FCGS – especially in cats that are unsuitable for long-term steroid use or surgical intervention. The product format was well-accepted by owners and may reduce treatment stress associated with oral medications.

The strengths of this study include its double-blind, randomized, placebo-controlled design, use of validated clinical scoring systems, and integration of both veterinary and owner-based assessments. However, the study is not without limitations. The small sample size (n = 16) likely limited statistical power and increased the risk of Type II error. Furthermore, the short treatment duration (28 days) may have been insufficient to capture the full therapeutic potential of n-3 PUFAs in a chronic, multifactorial condition such as FCGS. In addition, the use of coarse scoring systems (0–3 scales) may have lacked sensitivity to detect subtle clinical changes.

Looking ahead, future research should involve larger sample sizes, longer supplementation periods (e.g., 8–12 weeks), and a broader range of n-3 PUFA dosages to establish optimal treatment thresholds. More granular clinical tools, such as the SDAI and higher-resolution pain scales, are also recommended to enhance outcome sensitivity. Longitudinal studies evaluating the integration of PUFA supplementation with existing medical or surgical interventions may further elucidate its role in multimodal FCGS management.

In summary, this pilot study provides encoura-ging preliminary evidence that omega-3-enriched cat treats are a safe and potentially supportive strategy for managing FCGS-related discomfort. While the clinical effects were modest and not statistically signifi-cant, the observed trends, safety profile, and ease of administration justify further investigation into their long-term benefits and integration into standard care protocols for FCGS.

## DATA AVAILABILITY

Supplementary data can be made available from the corresponding author on request.

## AUTHORS’ CONTRIBUTIONS

PS, SP, PP, and WC: Conceptualization, research design, methodology, data analysis, investigation, data curation, writing of the original draft, and visualization. CS: Methodology and data analysis. PS: Validation and project administration. WC: Supervision and project administration. PS, SP, PP, CS, and WC: Review and editing of the manuscript. All authors have read and approved the final version of the manuscript.
